# Circulating adrenal 11-oxygenated androgens are associated with clinical outcome in endometrial cancer

**DOI:** 10.3389/fendo.2023.1156680

**Published:** 2023-05-23

**Authors:** Cylia Dahmani, Patrick Caron, David Simonyan, Véronique Turcotte, Jean Grégoire, Marie Plante, Chantal Guillemette

**Affiliations:** ^1^ Centre Hospitalier Universitaire de Québec (CHU de Québec) Research Center, Cancer Research Center (CRC) of Université Laval and Faculty of Pharmacy, Université Laval, Québec, QC, Canada; ^2^ Statistical and Clinical Research Platform, CHU de Québec Research Center, Québec, QC, Canada; ^3^ Gynecologic Oncology Service, CHU de Québec, and Department of Obstetrics, Gynecology, Reproduction, Faculty of Medicine, Université Laval, Québec, QC, Canada; ^4^ Canada Research Chair in Pharmacogenomics, Université Laval, Québec, QC, Canada

**Keywords:** endometrial cancer (EC), 11-oxygenated androgens, mass spectrometry - LC-MS/MS, recurrence, survival

## Abstract

**Context:**

Recent evidence support that androgens play an important role in the etiology of endometrial cancer (EC). Adrenal-derived 11-oxygenated androgens are highly potent agonists of the androgen receptor (AR), comparable to testosterone (T) and dihydrotestosterone (DHT) that have not been studied in the context of EC.

**Methodology:**

We studied a cohort of 272 newly diagnosed postmenopausal EC cases undergoing surgical treatment. Circulating concentrations of seven 11-oxygenated androgens including precursors, potent androgens and their metabolites were established in serum samples collected before and 1 month after surgery using a validated liquid chromatography tandem mass spectrometry method (LC-MS/MS). Free (unconjugated) and total (free + sulfate and glucuronide conjugates following enzymatic hydrolysis) were analyzed in relation to clinicopathological features, recurrence and disease-free survival (DFS).

**Results:**

Levels of 11-oxygenated androgens were weakly correlated to those of canonical androgens such as testosterone (T) and dihydrotestosterone (DHT), with no evidence of their association with clinicopathological features. Levels of 11-oxygenated androgens declined after surgery but remained higher in overweight and obese compared to normal weight cases. Higher levels of preoperative free 11-ketoandrosterone (11KAST) were associated with an increased risk of recurrence (Hazard ratio (HR) of 2.99 (95%CI=1.09-8.18); *P*=0.03). Postoperative free 11β-hydroxyandrosterone (11OHAST) levels were adversely associated with recurrence and DFS (HR = 3.23 (1.11-9.40); *P*=0.03 and 3.27 (1.34-8.00); *P*=0.009, respectively).

**Conclusion:**

11-oxygenated androgen metabolites emerge as potential prognostic markers of EC.

## Introduction

Endometrial cancer (EC) is the most common gynecological cancer in the Western world, predominantly affecting postmenopausal women (1). The majority of cases is diagnosed at an early stage and presents a favorable prognosis. Hysterectomy is the first line treatment for cases with localized tumors, however, a subset of patients experience recurrence. EC is a hormone-dependent cancer, in which sex steroid hormones, and particularly estrogens, play a major role.

Studies ascertained the relationships between circulating adrenal precursors, androgens and/or estrogens as well as germline variations in steroid-related biosynthesis pathways with the risk of EC (2). However, knowledge remains limited regarding the relation between circulating steroid levels and prognosis for EC patients. One study of 246 EC cases showed that higher preoperative serum levels of the estrogen precursor estrone-sulfate (E_1_-S) and estradiol (E_2_) metabolites were associated with recurrence after hysterectomy and shorter overall survival (3). Another study evidenced that lower plasma levels of androstenedione (A4), the precursor of E_1_, were associated with aggressive tumor characteristics and poor survival in 100 EC cases (4). In a limited series of 19 EC cases with shorter survival that died within 3 years after diagnosis, lower preoperative levels of E_1_-S as well as the adrenal derived androgen precursor dehydroepiandrosterone (DHEA) and its sulfated derivative DHEA-S were observed, compared to 19 EC cases with prolonged survival, with no evidence of differences for E_2_ between groups (5). After menopause, the adrenal glands secrete DHEA and A4 as the main precursors of potent androgen (AR) and estrogen (ER) receptor ligands, and their levels decline with age (6). Aromatization of androgens to estrogens is very active in the adipose tissues and as a result, body mass index (BMI) is directly correlated with E_1_ and E_2_ levels in postmenopausal women (3). Levels of androgens such as testosterone (T) and dihydrotestosterone (DHT) have also been shown to increase in EC cases, both in endometrioid and non-endometrioid diseases (3, 7). In addition to their aromatization to estrogens in multiple sites including adipose and uterine tissues (8–11), androgens and AR signaling pathway may play different roles in EC (12–14). Both oncogenic and tumor suppressive roles have been reported along with variable AR expression levels depending on the stage of disease (15, 16). In EC cell models, evidence suggests interference with the progestin signaling, promotion of cell proliferation and drug resistance, although with inconsistent results (17–20). These findings reinforce the need to examine whether additional androgens and AR-ligands influence EC prognosis.

Recent studies showed that circulating levels of non-canonical 11-oxygenated androgens originating primarily in the adrenal gland do not decline with age (21, 22). Their biosynthesis arises from the precursor 11β-hydroxyandrostenedione (11OHA4) and 11-ketoandrostenedione (11KA4) to produce 11-ketotestosterone (11KT), 11β-hydroxytestosterone (11OHT) and 11-ketodihydrotestosterone (11KDHT) with androgenic activity, representing additional AR ligands (23, 24). Their metabolites 11-ketoandrosterone (11KAST) and mainly 11β-hydroxyandrosterone (11OHAST) are also abundant in circulation of healthy men and women (21). In a recent publication that measured all seven of them using a validated mass spectrometry (MS) assay (21), 11-oxygenated androgens were associated with poor prognosis in a cohort of 1793 men with localized prostate cancer undergoing prostatectomy (25). Other studies using either non validated and validated assays (discussed in reference 21), reported few of these non-canonical 11-oxygenated androgens in a number of other clinical conditions, including androgen excess conditions, Cushing’s syndrome and congenital adrenal hyperplasia, all using validated MS methods (24, 26–29).

In this study, we sought to provide a detailed assessment of both free and total circulating levels of 11-oxygenated androgens in EC cases, measured in preoperative and postoperative blood specimens by MS, and establish their link with canonical androgens, BMI, poor prognostic features, recurrence and disease-free survival (DFS) after surgery. We studied a prospective cohort of 272 postmenopausal EC cases undergoing hysterectomy. Data exposed that circulating 11-oxygenated androgen metabolites are linked to adverse outcomes in EC cases, independent of established prognostic features, suggesting their role in EC progression.

## Materials and methods

### Study patients and data collection

All patients were recruited at The Hôtel-Dieu de Québec Hospital (Québec City, Canada), between 2002 and 2013. To be included in the study, women had to be postmenopausal, undergoing surgery for EC (hysterectomy and bilateral salpingo-oophorectomy), and not having taken hormone-replacement therapy (HRT) in the three weeks prior to surgery. Demographic information and anthropometric data were collected through a nurse-administered questionnaire, including HRT. A pathologist assessed histopathological characteristics of each hysterectomy specimen. The assembling and review of medical records was performed by one of the treating gynecologic oncologists (JG). Blood sampling were performed early morning the day of surgery and approximately one month later as part of a follow-up appointment. This cohort has also been described in more details previously (3). All participants provided a written informed consent for their participation in the study and the use of their specimens, and the protocol was evaluated and approved by the local Ethical Research Committee (CHUQc-UL #2012-993).

### Quantification of 11-oxygenated androgens by mass spectrometry

Quantification of circulating 11-oxygenated androgens was performed using a recently validated liquid chromatography tandem mass spectrometry (LC-MS/MS) assay using deuterated steroids as internal standard, as described in our previous work (21). Briefly, we used 200 µl of serum to quantify 11OHA4, 11KA4, 11OHT, 11KT, 11KDHT, 11OHAST and 11KAST, analyzed in a blinded fashion. Sample preparation consisted of a liquid-liquid extraction followed by chemical hydroxylamine derivatization (21). Prior to extraction, an enzymatic hydrolysis treatment was performed using β-glucuronidase/sulfatase overnight at 37C° to measure total 11-oxygenated androgens, corresponding to the sum of unconjugated and conjugated forms (sulfates + glucuronides) of steroids, as described (21). For measurement of free or unconjugated 11-oxygenated androgens, this hydrolysis step was omitted. Quality controls were included in each run, and coefficients of variation for each metabolite were <10%. Lower limits of quantification were 10 pg/mL and 20 pg/mL for free (unconjugated) and total (unconjugated and conjugated) fractions, respectively. Levels of canonical steroids assessed by LC-MS/MS were available from our previous study of the same EC cohort (3).

### Statistical analyses

Data were described as means, SD, confidence intervals (CI 95%), medians, and ranges, and categorical variables as frequencies and percentages. Categorical data were compared by Chi-square (χ^2^) tests or Fisher’s exact tests. Logarithmic transformation (with natural log values) was used for statistics to normalize skewed distribution of 11-oxygenated concentrations. Differences in 11-oxygenated androgen levels between groups were assessed using the analysis of covariance (ANCOVA). In pairwise comparisons of more than two groups, the Tukey-Kramer *post hoc* test was used. Variations in repeated measures of 11-oxygenated androgens between paired blood samples collected early morning were available for 186 cases (e.g. matched preoperative and postoperative samples) and analyzed using Wilcoxon signed rank test, as performed in our previous study similar in scope (3). Spearman correlations were estimated between steroids. The association between circulating 11-oxygenated androgens levels and outcomes (recurrence and survival) was estimated by univariable analyses using Kaplan-Meier and survival differences between groups compared with log-rank test. Multivariable analyses were performed by using Cox proportional-hazard models (HR). The statistical model was adjusted for age, BMI, histological type, myometrial invasion and SHBG levels, as performed in our previous study (3). Covariables were also specified in the legends of figures and tables. DFS was defined as the time from date of primary treatment (surgery) to the time of cancer recurrence/death. Two-sided *P* value less than 0.05 were considered statistically significant. Because of exploratory nature of study, all finding were considered hypothesis generating, adjustment for multiple testing correction was not performed. All statistical analyses were performed using SAS Statistical Software v.9.2. and graphs with GraphPad v.9.3.1.

## Results

### Characteristics of the prospective cohort of postmenopausal EC cases undergoing hysterectomy

**Figure 1:**
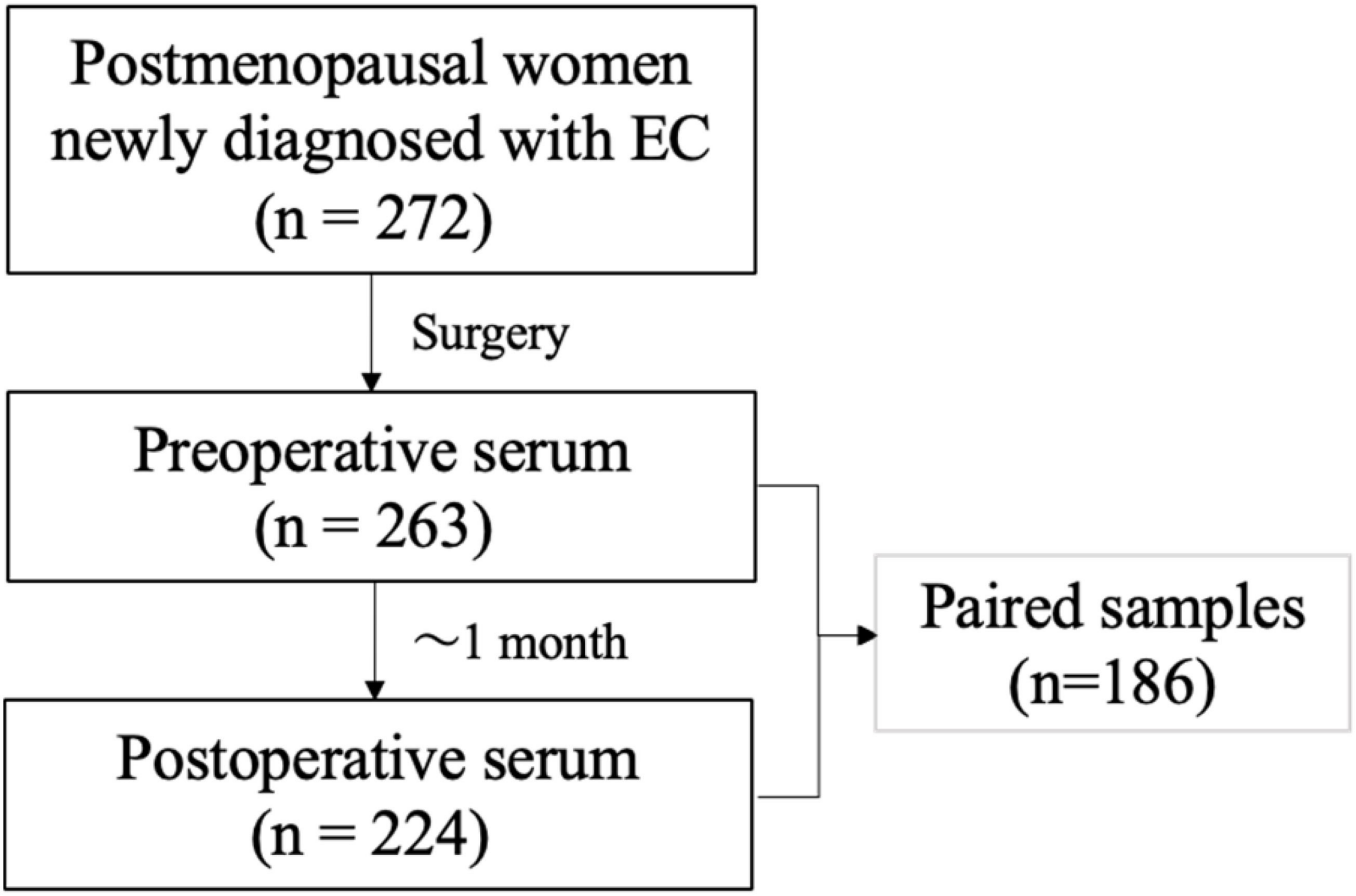
Flow chart of the study cohort and available serum specimens.

A prospective cohort of 272 postmenopausal women newly diagnosed with EC and all treated by hysterectomy was studied (**Figure 1**). The mean follow-up time was 60.5 months after hysterectomy and included thirty patients that experienced recurrence (11%). Serum samples were available prior to and after surgery for most cases, as indicated in **Figure 1**. Clinical features are depicted in **Table 1**. Cases were mostly type I adenocarcinoma (82%) and with 18% type II (histologically characterized by serous, clear cell, mucinous, or mixed carcinomas). Adverse histological features including myoinvasive tumors, the presence of metastatic node and lymph-vascular space invasion, were associated with DFS and recurrence but not BMI (**Table 2**). Poor prognosis based on preoperative risk classification was categorized as low (type I low grade G1 and G2) and high (type I high grade G3 and type II) risk groups, with most relapse cases occurring in the high-risk group (**Table 2**).

**Table 1:** Clinicopathological features of endometrial cancer (EC) cases undergoing hysterectomy.

Features		EC cases (n = 272)	
		Mean ± SD	
Age (yr)		65.5 ± 8.7	
Weight (kg)		75.0 ± 18.8	
Height (cm)		158.4 ± 6.4	
Follow-up (months)		60.3 ± 36.9	
5-year survival (%)		90.1	
5-year recurrence rate (%)		11.3	
		n (total of 272)	(%)
Body mass index (BMI)^1^			
Normal Weight		76	(28)
Overweight		77	(28)
Obese		117	(43)
Missing		2	(1)
Histological Type			
Type I		223	(82)
Type II		49	(18)
Grade			
1		103	(38)
2		102	(38)
3		66	(24)
Missing		1	(0)
Stage			
1		216	(79)
2		15	(6)
3		32	(12)
4		9	(3)
Myometrial invasion			
< 50%		203	(75)
≥ 50%		69	(25)
Lymph-vascular space invasion			
Absence		206	(76)
Presence		66	(24)
Presence of metastatic nodes			
No		241	(89)
Yes		31	(11)
Relapse after surgery^2^			
No		242	(89)
Yes		30	(11)

^1^Categories of BMI according to WHO Guidelines: normal weight: BMI<25 kg/m^2^, overweight: BMI between 25 and 30 kg/m^2^, and obese: BMI≥30 kg/m^2^.

^2^Clinicopathological features of endometrial cancer cases in relation to recurrence post-surgery are presented in **Table 2**.

**Table 2:** Clinicopathological features of EC cases undergoing surgery for curative intent in relation to recurrence and survival.

	Recurrence					Disease-Free Survival (DFS)				
Characteristics	No recurrence		Recurrence		Log-rank *P*	Alive		Recurred or Deceased		Log-rank *P*
	n=242	(%)	n=30	(%)		n=223	(%)	n=49	(%)	
BMI										
<25	66	(27)	10	(33)	0.5	61	(27)	15	(31)	0.6
25 to 30	67	(28)	10	(33)		61	(27)	16	(33)	
>30	107	(44)	10	(33)		99	(45)	18	(46)	
Missing	2	(1)	0	(0)		2	(1)	0	(0)	
Histological type										
Type I	208	(86)	15	(50)	**<0.0001**	192	(86)	31	(63)	**0.0004**
Type II	34	(14)	15	(50)		31	(14)	18	(47)	
Grade										
1	100	(41)	3	(10)	**0.0005**	93	(42)	10	(20)	**0.003**
2	90	(37)	12	(40)		83	(37)	19	(39)	
3	51	(21)	15	(50)		46	(21)	20	(41)	
Missing	1	(1)	0	(0)		1	(0)	0	(0)	
FIGO stage										
1	203	(84)	13	(44)	**<0.0001**	192	(86)	24	(49)	**<0.0001**
2	14	(6)	1	(3)		12	(5)	3	(6)	
3	20	(8)	12	(40)		15	(6)	17	(35)	
4	4	(2)	4	(13)		3	(1)	5	(10)	
Invasion of myometrium										
< 50%	190	(79)	13	(43)	**<0.0001**	177	(79)	26	(53)	**0.0002**
≥ 50%	52	(21)	17	(57)		46	(21)	23	(47)	
Lymph-vascular space invasion (LVSI)										
Absence	191	(79)	15	(50)	**<0.0006**	179	(80)	27	(55)	**0.0004**
Presence	51	(21)	15	(50)		44	(20)	22	(45)	
Metastatic nodes										
No	221	(91)	20	(67)	**<0.0001**	206	(92)	35	(71)	**0.0002**
Yes	21	(9)	10	(33)		17	(8)	14	(29)	
Poor prognosis^1^										
Low risk	187	(77)	13	(43)	**<0.0001**	173	(78)	27	(55)	**0.002**
High risk	55	(23)	17	(57)		50	(22)	22	(49)	
Overall survival^2^										
Alive	223	(92)	9	(30)	**<0.0001**					
Deceased	18	(8)	21	(70)						

^1^Risk of poor prognosis is categorized as low risk corresponding to type I (TI) with low-grade G1 and G2 whereas TI-G3 and TII are considered as high risk. ^2^Overall survival (all-cause mortality). P values are derived from a chi-square test and significant differences are indicated in bold.

### Circulating levels of 11-oxygenated androgens in EC cases

**Figure 2:**
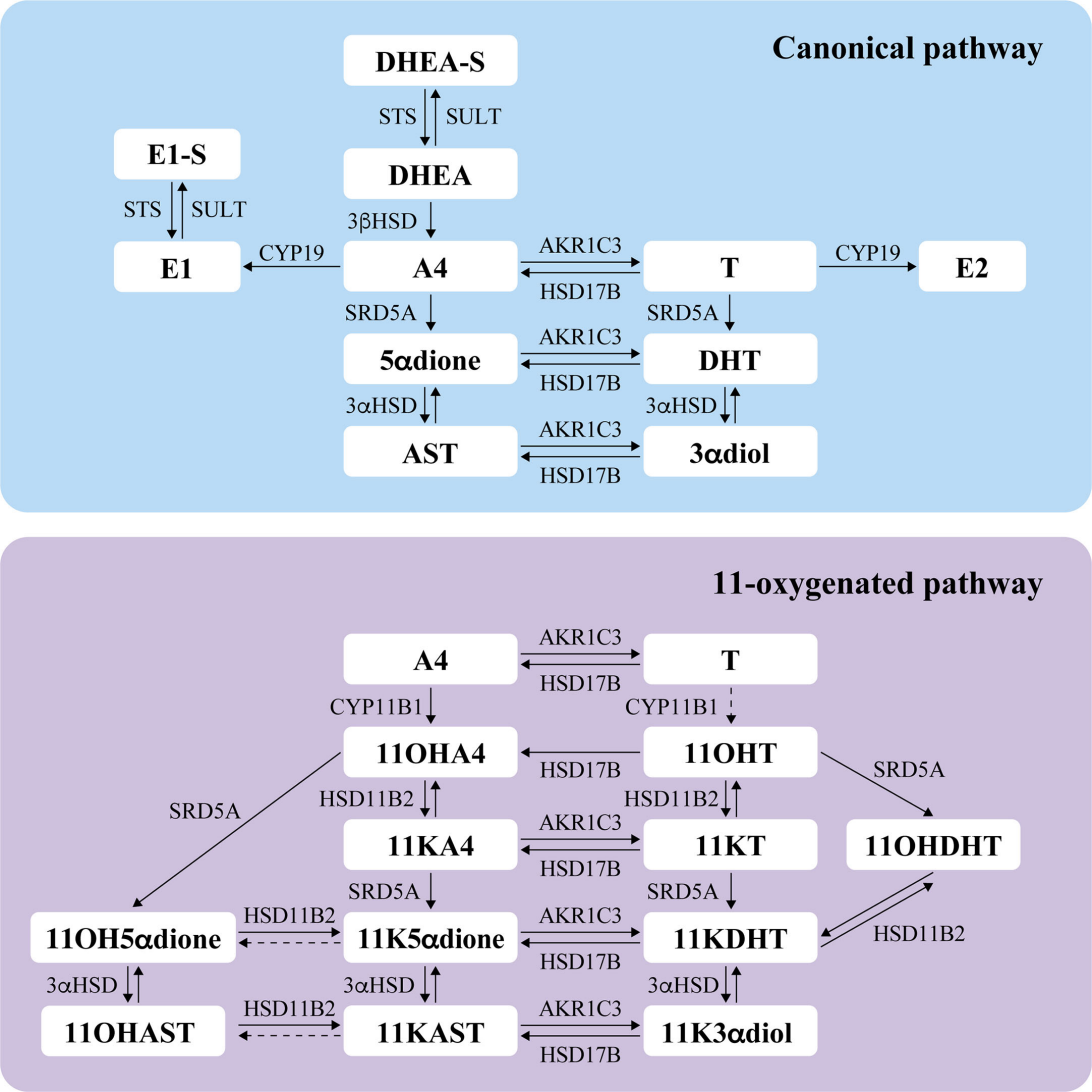
Schematic overview of steroidogenesis depicting the canonical and 11-oxygenated androgen pathways, based on data from the literature (23, 24, 29–38). Steroids assessed in this study are indicated in bold. The dotted arrow between T and 11OHT indicates an unfavorable reaction; A4 is the preferred substrate of CYP11B1 compared to T (30). The dotted arrows for the conversion of 11KAST to 11OHAST and 11K5αdione to 11OH5αdione represent biotransformation inconsistently reported in the literature (33, 34, 36, 39). DHEA, dehydroepiandrosterone; DHEA-S, dehydroepiandrosterone sulfate; A4, androstenedione; 5αdione, 5α-androstanedione; AST, androsterone; T, testosterone; DHT, 5α-dihydrotestosterone; 3αdiol, 5α-androstane-3α,17β-diol; E1, estrone; E1-S, estrone sulfate; E2, estradiol; 11OHA4, 11-hydroxyandrostenedione; 11KA4, 11-keto-androstenedione; 11OHT, 11-hydroxytestosterone; 11KT, 11-keto-testosterone; 11OHDHT, 11-hydroxydihydrotestosterone; 11KDHT, 11-keto-dihydrotestosterone; 11OH5αdione, 11β-hydroxy-5α-androstanedione; 11K5αdione, 11-keto-5α-androstanedione; 11OHAST, 11-hydroxyandrosterone; 11KAST, 11-keto-androsterone; 11K3αdiol, 11-keto-5α-androstane-3α,17β-diol. STS, steroid sulfatase; SULT, sulfotransferase; 3βHSD, 3β–hydroxysteroid dehydrogenase; SRD5A, 5α reductase; 3αHSD, 3α-hydroxysteroid dehydrogenase; AKR1C3, aldo-keto reductase family 1 member C3 (also known as 17β-hydroxysteroid dehydrogenase type 5); HSD17B, 17β-hydroxysteroid dehydrogenase; CYP19, aromatase; CYP11B1, cytochrome P450 11β-hydroxylase member 1; HSD11B2, 11β-hydroxysteroid dehydrogenase type 2.

**Figure 3:**
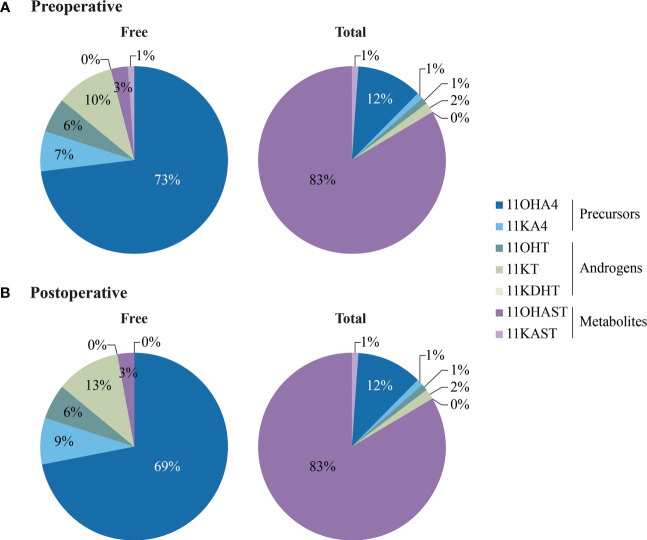
Pie charts showing the distribution of free (unconjugated) and total (unconjugated+ conjugated) 11-oxygenated androgens in preoperative **(A)** and postoperative **(B)** serums of EC cases. Steroid concentrations are presented in **Table 4**.

**Figure 4:**
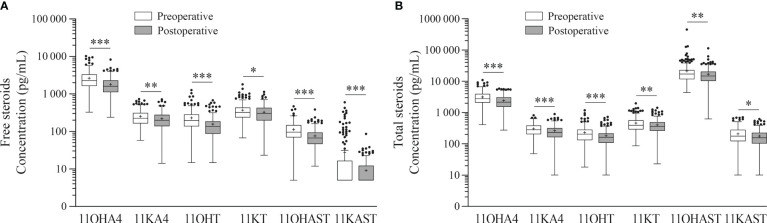
Comparison between preoperative and postoperative circulating levels of 11-oxygenated androgens. Free (unconjugated) **(A)** and total (free + conjugated (sulfates + glucuronides)) **(B)** 11-oxygenated androgens are depicted. Preoperative (white boxes) and postoperative (gray boxes) 11-oxygenated steroids were measured in serums from paired preoperative and postoperative paired samples of 186 EC cases. Box plot depicts 25-75 percentile and whiskers with the median are shown as solid lines and mean shown as +. Levels of 11-oxygenated androgens were log transformed and adjusted for age and BMI for statistics. * *P*< 0.05. ** *P*< 0.01. *** *P*< 0.001.

Seven 11-oxygenated androgens were measured by MS in serum samples collected early morning, the day of surgery and one month after. Steroids measured included two 11-oxygenated precursors (11OHA4 and 11KA4), full agonists 11KT and 11KDHT, partial agonist 11OHT, and their metabolites 11OHAST and 11KAST (**Figure 2**). For each steroid, free (unconjugated) and total (free + conjugated) 11-oxygenated androgens were reported. The majority of 11-oxygenated androgens were detected above LLOQ, except free 11KAST detected in 49% preoperative (130/263) and 31% postoperative (70/224) serums (**Table 3**). When limited to 186 cases with paired preoperative and postoperative specimens, similar observations were noted. Another exception is the AR agonist 11KDHT that was detected in 13% preoperative serums (34/263) as total 11KDHT, and not detected above LLOQ for free/unconjugated 11KDHT. In cases with detectable total 11KDHT, we also observed higher free preoperative 11OHAST compared to cases with no detectable 11KDHT (median levels of 115 vs 92 pg/ml; *P* = 0.018), whereas levels of 11KAST were not significantly different. The unconjugated adrenal precursor 11OHA4 was the predominant 11-oxygenated androgen in circulation of EC cases before surgery, representing 73% of all measured 11-oxygenated androgens while free 11OHT and 11KT represented 16% (**Figure 3A**). The levels of the free metabolites 11OHAST and 11KAST were less abundant (4% of all measured 11-oxygenated androgens). When assessing the total fraction comprising free and conjugated steroids, levels of the most abundant total 11OHAST represented 83% of all 11-oxygenated androgens measured. In postoperative serums, a similar distribution of 11-oxygenated androgens was observed (**Figure 3B**). Compared to preoperative levels of free 11-oxgenated androgens, those assessed after surgery for the same cases were reduced by 11 to 33% (*P* < 0.001) (**Table 4**; **Figure 4**), a finding replicated in the analysis of all samples.

**Table 3:** Percent of detection (%) of 11-oxygenated androgens measured by mass spectrometry in preoperative and postoperative serums of endometrial cancer cases.

Steroids (pg/mL)	Preoperative (%) (n=263)	Postoperative (%) (n=224)
Free		
11OHA4	100	100
11KA4	100	100
11OHT	100	100
11KT	100	100
11KDHT	n.d.	n.d.
11OHAST	100	100
11KAST	49	31
Total		
11OHA4	100	100
11KA4	100	99
11OHT	99	99
11KT	100	100
11KDHT	13	n.d.
11OHAST	100	100
11KAST	99	99

Detection was defined as levels of 11-oxygenated androgens above the lower limit of quantification (LLOQ) of 10 pg/mL for free 11-oxygenated androgens and 20 pg/mL for total 11-oxygenated androgens. n.d. below LLOQ in all cases and therefore, 11KDHT was not pursued in further analyses.

Free = unconjugated 11-oxygenated androgens; Total = unconjugated + conjugated 11-oxygenated.

**Table 4:** Comparison of median (10^th^ and 90^th^ percentile) levels of steroids in paired preoperative and postoperative serums from 186 endometrial cancer cases.

	Preoperative		Postoperative		Median variation^1^	Fold change
Steroids (pg/mL)	Median	10^th^ and 90^th^ percentile	Median	10^th^ and 90^th^ percentile	(Post vs Pre)	(Post vs Pre)
Free						
11OHA4	2240	1200 - 4440	1560	742 - 2910	**-755**	**0.70**
11KA4	217	121 - 419	194	109 - 348	**-20**	**0.89**
11OHT	193	89 - 394	129	62 - 270	**-62**	**0.67**
11KT	323	161 - 644	288	139 - 541	**-34**	**0.89**
11OHAST	94	47 - 184	66	34 - 121	**-31**	**0.71**
11KAST	5	5 - 94	5	5 – 17	**0**	1.00
Total						
11OHA4	2775	1670 - 5260	2135	1150 - 3790	**-645**	**0.77**
11KA4	270	124 - 464	226	128 - 445	**-24**	**0.84**
11OHT	201	84 - 402	155	71 - 305	**-41**	**0.92**
11KT	378	220 - 773	349	173 - 694	**-28**	**0.92**
11OHAST	16750	8610 - 31600	14800	7290 - 30500	**-1200**	**0.88**
11KAST	171	68 - 362	153	69 - 287	**-13**	**0.89**
SHBG (nmol/L)	65	31 - 125	65	30 - 114	-1.4	1.00

Median values above levels of detection and ranges (10^th^ and 90^th^ percentile) are displayed.

^1^Variations were established using Wilcoxon signed rank test for paired data. Fold changes were calculated upon median of each group. Bold = P < 0.001.

Levels of free 11-oxygenated androgens were weakly correlated to BMI (r ≤ 0.25), except for 11OHT that displayed slightly higher correlation values (r = 0.38 and 0.35 for preoperative and postoperative levels, respectively (*P* < 0.001) (**Supplementary Table 1**). Using BMI categories, we also noted that levels of preoperative 11-oxygenated androgens and most particularly 11OHT, were higher for overweight (1.1 to 2.1-fold; BMI 25 to 29.9 kg/m^2^ *; P* < 0.05) and obese EC cases (1.2 to 2.1-fold; BMI > 30 kg/m^2^ *; P* < 0.05), compared to those with normal weight (BMI <25 kg/m^2^) (**Supplementary Table 2**). After surgery, most 11-oxygenated androgens remained individually higher for overweight (1.1-1.5-fold; BMI 25 to 29.9 kg/m^2^; *P* < 0.05) and obese EC cases (1.2 to 1.6-fold; BMI > 30 kg/m^2^; *P* < 0.05), compared to those with normal weight (BMI <25 kg/m^2^) (**Supplementary Table 3**).

### Correlation between circulating 11-oxygenated androgens and canonical steroids, before and after hysterectomy

**Figure 5:**
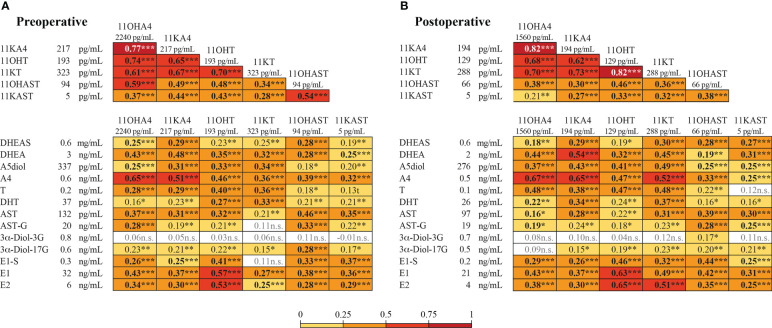
Correlation between preoperative and postoperative levels of 11-oxygenated androgens and canonical steroids in paired preoperative and postoperative samples from 186 EC cases. Heatmap of free 11-oxygenated androgens and canonical steroids in preoperative **(A)** and postoperative **(B)** serums. Spearman r values are shown in the box. Significant correlations are shaded as per the colors in the key. Levels of 11-oxygenated steroids (this study) and of canonical steroids (previously reported (3)) are given on the side of heatmaps. Significant data (*P*< 0.05) are indicated in bold. * *P*< 0.05. ** *P*< 0.01. *** *P*< 0.001.

Preoperative levels of 11-oxygenated androgens were strongly correlated (**Figure 5A**). The strongest correlations were observed for precursors 11OHA4 and 11KA4 and the androgenic 11OHT and 11KT (r = 0.61-0.77; *P* < 0.001). Levels of 11OHAST and 11KAST metabolites displayed weak to moderate correlations with the 11-oxygenated precursors and the androgenic 11KT (r = 0.28-0.49; *P* < 0.001). These correlations remained significant after surgery, although they were weaker between precursors and 11-oxygenated androgens metabolites (**Figure 5B**). We further assessed the relationships between levels of 11-oxygenated androgens and canonical steroids measured in our previous study of the same cohort, including abundant precursor derived from the adrenal gland such as DHEA (3). In preoperative serums, levels of 11-oxygenated androgens were weakly to moderately correlated to those of canonical adrenal precursors DHEA and A4 (r = 0.25 to 0.48; *P* < 0.05). The strongest correlations were noted between A4 and 11OHA4 (r = 0.65; *P* < 0.001) and 11KA4 (r = 0.51; *P* < 0.001). The canonical AR ligands T and DHT displayed modest correlations with the AR agonist 11KT (r = 0.36 and 0.33, respectively; *P* < 0.001). Furthermore, estrone (E_1_) and estradiol (E_2_) were correlated to partial AR agonist 11OHT in both preoperative (r = 0.57 and 0.53, respectively; *P* < 0.001) and postoperative (r = 0.63 and 0.65, respectively; *P* < 0.001) serums. Following surgery, strongest correlations were observed between the AR agonist 11KT and levels of A4, T, E_1_ and E_2_ (r values ranging from 0.48 to 0.52, respectively; *P* < 0.001) (**Figure 5B**).

### Levels of 11-oxygenated androgens were not associated with clinicopathological features

None of the 11-oxygenated androgens, including for preoperative total 11KDHT or free 11KAST detected in a subset of cases, were significantly associated with clinicopathological features, including histological type, grade, stage, myometrial and lymph-vascular space invasion (not shown). Cases with type II EC presented lower levels of postoperative potent androgens 11KT (by 27%; *P* = 0.005) and 11OHT (by 22%; *P* = 0.03), whereas no differences were seen for cases diagnosed with type I (**Supplementary Table 4**). Lower levels of free 11KT (17%; *P* = 0.04) were also observed for postoperative serums of cases in the high-risk group, compared to those in the low risk group (**Supplementary Figure 1**).

### Levels of 11-oxygenated androgens were associated with clinical outcomes

**Figure 6:**
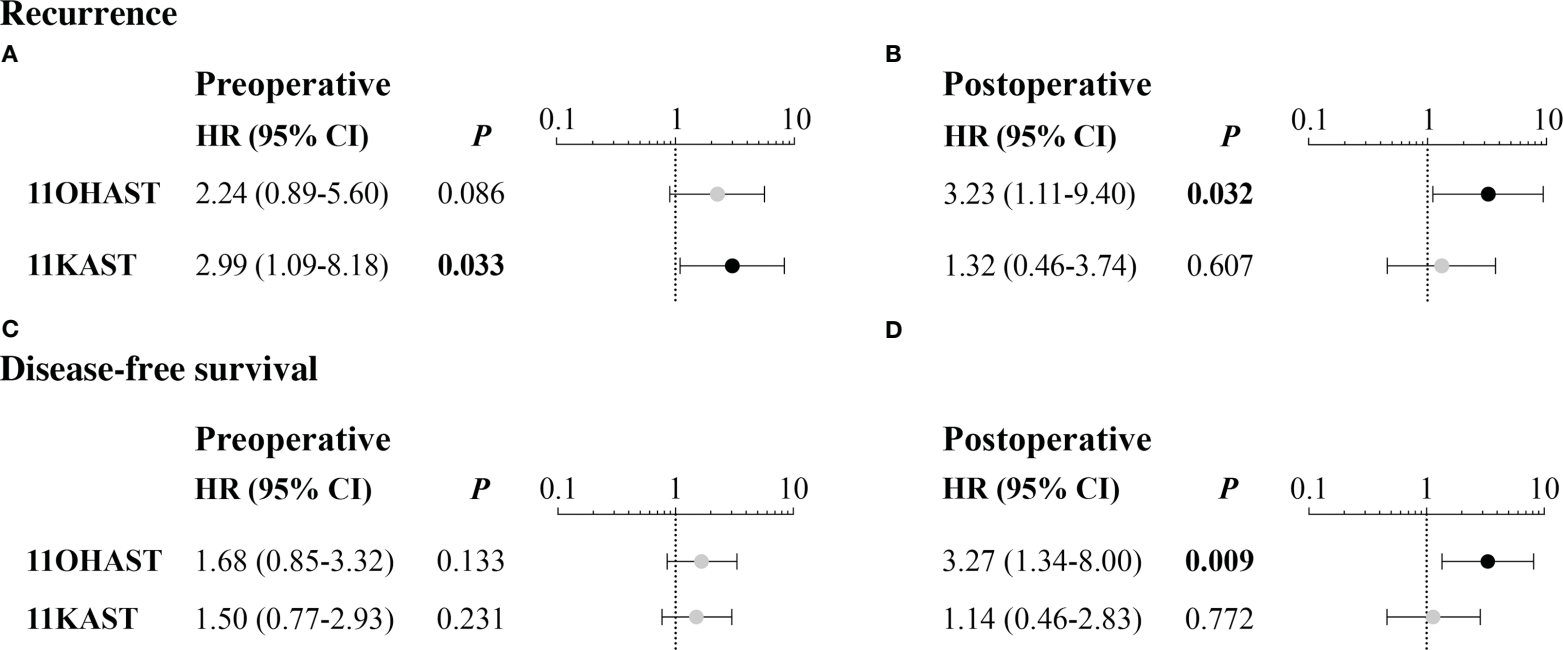
Risk of recurrence and disease-free survival in relation to free preoperative and postoperative levels of 11-oxygenated androgens. Hazard ratios (HR) were calculated using Cox regression for all available follow-up and comparing hormone categories separated upon median, with adjustment for age, BMI, histological type and myometrial invasion and SHBG levels, and as performed for canonical steroids in a previous report (3). Only significant relationships are depicted for recurrence **(A, B)** and disease-free survival **(C, D)** whereas all results of these analyses are presented in **Supplementary Tables 5, 6**.

In Kaplan-Meier analyses, a shorter time to recurrence was significant for EC cases with higher preoperative free 11KAST (*P* = 0.005) and 11OHAST (*P* = 0.04) (**Supplementary Figure 2A-C**). In multivariable analyses with the fully adjusted model, an increased risk of recurrence was observed for free 11OHAST measured before surgery (HR = 2.24 (0.89-5.60; *P* = 0.09) that reached significance for levels measured after surgery (HR = 2.99 (95% CI=1.09-8.18; *P* = 0.03) (**Figure 6**, **Supplementary Table 5**). A significant increased risk of recurrence was also observed for preoperative levels of free 11KAST detected at significantly lower levels than 11OHAST, with an HR value of 3.23 (95% CI = 1.11-9.40; *P* = 0.03), but was not significant after surgery, potentially due to a significant proportion of cases for which 11KAST remained undetectable (**Figure 6**). Kaplan-Meier revealed that high postoperative levels of free 11OHAST were associated with poorer DFS (*P* = 0.02), which remained significant in multivariable analyses (HR=3.27 (95 CI%=1.34-8.00; *P* = 0.009) (**Supplementary Figure 2D**, **Supplementary Table 6**). For total preoperative 11KDHT, despite the low number of cases with detectable levels (n=34), a trend towards a higher risk of poorer DFS was observed in multivariable analyses (HR = 2.20 (0.99-4.87; *P*=0.05), with no relationship to recurrence.

## Discussion

This study provides the first comprehensive profiling of free and total circulating 11-oxygenated androgens in postmenopausal women diagnosed with EC undergoing surgical treatment. The main observation relates to their link to an increased risk of recurrence and poorer DFS, suggesting that exposition to this novel class of androgens may sustain EC progression and serve as prognostic indicators and support the relevance of profiling these additional AR ligands. Findings contrast with those of our previous study focused on canonical androgens such as T and DHT that did not demonstrate a relationship with clinical outcomes, despite higher T and DHT levels measured in circulation of EC cases compared to healthy donors (3). We also provide evidence that circulating levels of 11-oxygenated androgens weakly correlate to known prognostic factors, further supporting their independent prognostic significance.

In postmenopausal women with EC, the adrenal precursor 11OHA4 was the predominant unconjugated 11-oxygenated androgen (median range 2.24 ng/ml or 7.41 nM) in preoperative serums, with levels comparable to those of DHEA observed in the same cohort (median levels of 2.62 ng/mL or 9.10 nM) (3). However, circulating levels of 11KDHT remained undetectable in circulation before and after surgery for most women, with only 13% of EC cases displaying detectable total 11KDHT in preoperative samples, preventing us to fully appreciate its association with disease characteristics and outcome. The 11OHT and 11KT with androgenic activity were detected at a median range of 193–223 pg/mL (0.74 nM) comparable to those of T (240 pg/mL or 0.83 nM) previously reported in the same EC cohort (3). This observation supports similar concentrations of circulating canonical and non-canonical androgens in postmenopausal women. No study evaluated 11-oxygenated metabolites 11KAST and 11OHAST in postmenopausal women, preventing us to compare levels across cohorts. Their higher levels and particularly those of the abundant 11OHAST were associated with an increased risk of relapse after surgery and shorter DFS. A recent study reported minimal or no androgenic bioactivity for these 11-oxygenated metabolites (40). In multivariate model adjusted for age, BMI and known prognostic variables, higher postoperative levels of 11OHAST were significantly associated with adverse outcomes and may reflect an enhanced conversion of 11-oxygenated AR agonists by peripheral tissues and/or residual and disseminated tumoral cells, favoring EC progression and relapse. In support, cases presenting higher total preoperative levels of the most potent 11KDHT agonist presented a 2.2-fold higher risk of poorer DFS that did not reached significance (*P* = 0.05). However, this finding should be interpreted with caution due to the low number of patients.

The levels of all 11-oxygenated androgens significantly declined one month after surgery, supporting that EC tumors and/or uterus and the surrounding organs removed by hysterectomy, meaningfully contribute to the activation of adrenal 11-oxygenated androgens. This is sustained by lower circulating levels of the 11KT with high androgenic potency in postoperative serums of type II cases and those in the high-risk category with poorer prognosis, compared to preoperative levels. Furthermore, previous reports showed that the main enzymes involved in the biotransformation of 11-oxygenated androgens are expressed in EC tumors, such as SRD5A, HSD11B2 and AKR1C3 (13, 41, 42). Levels of 11-oxygenated androgens were increased in obese and overweight compared to normal weight cases but were weakly linked to BMI (r ≤ 0.25), except for 11OHT which displayed a moderate association (r ~0.35). We also observed that the levels of 11OHT remained superior after surgery, suggesting that the adipose tissue may contribute and consistent with the expression of HSD11B1 involved in the conversion of 11KT to 11OHT (23).

Strengths of this study include measurement of a novel class of non-canonical androgens with a robust validated bioanalytical method based on mass spectrometry, comparison of their levels to those of canonical androgens assessed in the same cohort also measured by mass spectrometry, and investigations of their link with clinical outcomes based on a large cohort of newly diagnosed postmenopausal EC cases undergoing hysterectomy with extended follow-up, detailed clinicopathological parameters, and not using HRT. These findings are however limited by the small number of recurrent cases whereas no adjustments for multiple testing were performed given the exploratory nature of our investigation. A replication of our findings is warranted. At the tissue levels, measures of these novel androgens in EC tumors collected at surgery would help investigate whether circulatory levels reflect tissue steroid levels. These data will also help support their prognostic potential. Nonetheless, we hope that these initial findings will prompt other groups to investigate 11OHAST and 11KAST with more depth by adding these inactive 11-oxygenated metabolites to their analysis of the steroidome in EC and other clinical conditions, and using validated methods.

The findings that higher levels of 11-oxygenated androgen metabolites, 11OHAST and/or 11KAST measured before and after hysterectomy, were linked to an increased risk of recurrence and poorer DFS, provide insights for future research investigating the role of 11-oxygenated androgens in EC cancer, as they might serve as prognostic indicators. Further research is necessary to understand the effect of excess adrenal 11-oxygenated androgens in promoting EC progression.

## Data availability statement

The original contributions presented in the study are included in the article/**Supplementary Material**. Further inquiries can be directed to the corresponding author.

## Ethics statement

The studies involving human participants were reviewed and approved by CHU de Québec - Université Laval ethics committee. The patients/participants provided their written informed consent to participate in this study.

## Author contributions

Study concept and design: CG. Patient recruitment and clinical data: MP, JG. Conducted experiments and mass spectrometry: PC, VT. Statistical analyses: CD, DS, CG. Drafting of the manuscript: CD, CG. Critical revision of the manuscript for important intellectual content: All authors. Obtaining funding: CG. All authors contributed to the article and approved the submitted version.
