# Supplementary material for: Circulating adrenal 11-oxygenated androgens are associated with clinical outcome in endometrial cancer
Source: Front Endocrinol (Lausanne). 2023 May 23;14:1156680. doi: 10.3389/fendo.2023.1156680 (PMC10242140; doi:10.3389/fendo.2023.1156680)
Supplement: Supplementary file 1 [file DataSheet_1.pdf]

Circulating adrenal 11-oxygenated androgens are associated with clinical outcome in endometrial cancer.

Cylia Dahmani [1], Patrick Caron[1], David Simonyan[2], Véronique Turcotte[1], Jean Grégoire[3], Marie Plante[3] and Chantal Guillemette[1,4]

Supplementary Material:

Supplementary Table 1: Correlations between circulating 11-oxygenated androgens and BMI in preoperative and postoperative serum of EC cases.
Supplementary Table 2: Preoperative levels of 11-oxygenated androgens in endometrial cases by BMI categories.
Supplementary Table 3: Postoperative levels of 11-oxygenated androgens in endometrial cases by BMI categories.
Supplementary Table 4: Preoperative and postoperative levels of 11-oxygenated androgens in type I and II endometrial cases.
Supplementary Table 5. Risk of EC recurrence in relation to levels of 11-oxygenated androgens.
Supplementary Table 6. Disease-free survival (DFS) in relation to 11-oxygenated androgens
Supplementary Figure 1: Postoperative levels of 11-oxygenated androgens were associated with poor prognosis in endometrial cancer cases.
Supplementary Figure 2: Higher levels of 11-oxygenated androgens were associated with shorter time to recurrence and disease-free survival in EC cases.

Supplementary Table 1. Correlations between circulating 11-oxygenated androgens and BMI in preoperative and postoperative serum of EC cases.

r values	Preoperative	Postoperative
**Free**		
11OHA4	0.25***	0.17*
11KA4	0.16**	0.09
11OHT	**0.38*****	**0.35*****
11KT	0.19**	0.22**
11OHAST	0.16*	0.21**
11KAST	0.12	0.07
**Total**		
11OHA4	0.16**	0.11
11KA4	0.04	0.02
11OHT	**0.39*****	**0.44*****
11KT	0.27***	0.23**
11OHAST	0.12	0.13
11KAST	0.20**	0.26***

Data are shown as Spearman Correlation Coefficients. Moderate correlations are highlighted in bold ($r \geq 0.35$). ***$P < 0.001$; **$P < 0.01$; *$P < 0.05$.

Supplementary Table 2. Preoperative levels of 11-oxygenated androgens in endometrial cases by BMI categories.

Steroids (pg/mL)	Normal weight BMI < 25 kg/m^2 (n=73)		Overweight BMI = 25-29.9 kg/m^2 (n=73)		Obese BMI > 30 kg/m^2 (n=116)		Fold Changes: Overweight vs Normal	Fold Changes: Obese vs Normal	Fold Changes: Obese vs Overweight
**Free**									
	Median	10th – 90th	Median	10th – 90th	Median	10th – 90th			
11OHA4	1860.0	1110.0 – 3190.0	2530.0	863.0 – 4820.0	2540.0	1380.0 – 4820.0	1.36	1.37***	1.00
11KA4	200.0	118.0 – 316.0	238.0	121.0 – 473.0	238.0	136.0 – 416.0	1.19*	1.19*	1.00
11OHT	155.0	77.2 – 276.0	198.0	99.8 – 389.0	227.0	130.0 – 398.0	1.28**	1.46***	1.15
11KT	278.0	161.0 – 461.0	338.0	165.0 – 584.0	342.0	179.0 – 672.0	1.22	1.23*	1.01
11OHAST	87.1	46.1 – 159.0	93.9	47.3- – 181.0	109.0	55.0 – 201.0	1.08	1.25*	1.16
11KAST	5.0	5.0 – 17.6	10.5	5.0 – 55.1	10.4	5.0 – 99.9	2.10	2.08	0.99
**Total**									
11OHA4	2570.0	1420.0 – 4090.0	2855.0	1490.0 – 5470.0	2980.0	1800.0 – 5460.0	1.11*	1.16**	1.04
11KA4	254.0	142.0 – 442.0	306.0	128.0 – 513.0	280.0	139.0 – 466.0	1.20	1.10	0.92
11OHT	134.0	60.50 – 269.0	208.5	83.2 – 426.0	238.0	121.0 – 403.0	1.56***	1.78***	1.14*
11KT	331.0	220.0 – 568.0	398.5	242.0 – 779.0	420.0	252.0 – 818.0	1.20*	1.27***	1.05
11OHAST (ng/mL)	15.40	8.61 – 25.5	16.4	8.9 – 36.50	17.90	8.8 – 37.6	1.06	1.16*	1.09
11KAST	136.0	54.50 – 292.0	179.5	69.7 – 466.0	197.0	93.5 – 405.0	1.32	1.45*	1.10
SHBG (nmol/L)	92.7	45.0 – 155.0	69.5	37.0 – 132.1	46.9	28.3 – 105.2	0.75	0.51***	0.68**

Data are shown as median (10th – 90th range). Fold changes were calculated using the median. ***$P < 0.001$; **$P < 0.01$; *$P < 0.05$.
Data were log-transformed and adjusted for age for statistical analyses. BMI: Body mass index

Supplementary Table 3. Postoperative levels of 11-oxygenated androgens in endometrial cases by BMI categories.

Steroids (pg/mL)	BMI categories: Normal weight BMI < 25 kg/m² (n=73)			Overweight BMI = 25-29.9 kg/m² (n=73)			Obese BMI > 30 kg/m² (n=116)			Fold Changes: Overweight vs Normal	Obese vs Normal	Obese vs Overweight
**Free**												
	Median	10th	90th	Median	10th	90th	Median	10th	90th			
11OHA4	1360.0	577.0	2640.0	1695.0	873.0	3530.0	1660.0	915.0	3280.0	1.25*	1.22*	0.98
11KA4	169.0	104.0	348.0	201.0	118.0	402.0	201.0	114.0	341.0	1.19	1.19	1.00
11OHT	94.1	42.0	207.0	142.5	70.4	272.0	153.0	83.9	286.0	1.51***	1.63***	1.07
11KT	235.0	122.0	518.0	308.5	157.0	541.0	332.0	147.0	558.0	1.31	1.41*	1.08
11OHAST	60.9	26.6	94.0	67.3	34.0	147.0	76.0	39.0	132.0	1.10	1.25*	1.13
11KAST	5.0	5.0	14.5	5.0	5.0	17.1	5.0	5.0	21.1	1.00	1.00	1.00
**Total**												
11OHA4	1990.0	923.0	3180.0	2445.0	1450.0	4600.0	2160.0	1180.0	4580.0	1.23*	1.09	0.88
11KA4	226.0	110.0	447.0	245.0	136.0	461.0	230.0	135.0	445.0	1.08	1.02	0.94
11OHT	109.0	51.90	215.0	167.0	81.50	306.0	186.0	106.0	348.0	1.53***	1.71***	1.11
11KT	306.0	164.0	562.0	361.0	210.0	694.0	389.0	219.0	709.0	1.18	1.27**	1.08
11OHAST (ng/mL)	12.8	6.27	27.50	14.5	8.1	28.2	15.90	7.64	32.40	1.13	1.24	1.10
11KAST	127.0	49.20	259.0	149.0	71.0	269.0	178.0	83.5	379.0	1.17	1.40**	1.19
SHBG (nmol/L)	87.3	40.7	150.9	66.2	35.1	112.6	48.4	25.5	93.2	0.80	0.54***	0.67*

Data are shown as median (10th – 90th range). Fold changes were calculated using the median. ***$P < 0.001$; **$P < 0.01$; *$P < 0.05$.
Data were log-transformed and adjusted for age for statistical analyses. BMI: Body mass index

Supplementary Table 4. Preoperative and postoperative levels of 11-oxygenated androgens in type I and II endometrial cases.

	Preoperative							Postoperative						
Steroids (pg/mL)	Type I (n= 213)			Type II (n= 48)			FC	Type I (n=187)			Type II (n= 35)			FC
Free														
	Median	10th	– 90th	Median	10th	– 90th		Median	10th	– 90th	Median	10th	– 90th	
11OHA4	2310.0	1200.0	– 4440.0	2180.0	1370.0	– 4130.0	0.94	1640.0	783.0	– 3100.0	1510.0	873.0	– 3160.0	0.92
11KA4	223.0	125.0	– 419.0	244.0	135.0	– 425.0	1.09	198.5	109.0	– 348.0	197.5	105.0	– 402.0	0.99
11OHT	205.0	99.80	– 378.0	175.0	88.5	– 348.0	0.85	140.0	71.0	– 278.0	87.95	54.3	– 257.0	0.63*
11KT	328.0	170.0	– 634.0	315.0	133.0	– 567.0	0.96	323.5	147.0	– 563.0	202.0	132.0	– 464.0	0.62**
11OHAST	95.3	53.1	– 183.0	99.9	44.2	– 228.0	1.05	66.8	36.9	– 122.0	67.60	31.3	– 167.0	1.01
11KAST	5.0	5.0	– 45.9	10.6	5.0	– 70.6	2.11	5.0	5.0	– 18.0	5.00	5.0	– 16.8	1.00
Total														
11OHA4	2785.0	1550.0	– 5260.0	2720.0	1680.0	– 5110.0	0.98	2155.0	1140.0	– 3930.0	2425.0	1260.0	– 4600.0	1.13
11KA4	279.5	139.0	– 486.0	287.0	122.0	– 442.0	1.03	230.5	131.0	– 445.0	235.5	114.0	– 481.0	1.02
11OHT	208.0	92.2	– 399.0	159.0	88.2	– 311.0	0.76	169.0	83.90	– 317.0	125.0	62.3	– 278.0	0.74*
11KT	391.5	233.0	– 757.0	380.0	220.0	– 746.0	0.97	371.5	186.0	– 700.0	279.5	169.0	– 579.0	0.75
11OHAST (ng/mL)	17.1	8.9	– 29.8	16.4	7.64	– 50.7	0.96	14.7	7.2	– 28.7	18.30	8.12	– 37.3	1.24
11KAST	178.5	70.9	– 374.0	161.5	52.2	– 412.0	0.90	163.5	74.9	– 327.0	141.5	59.1	– 307.0	0.87
SHBG (nmol/L)	63.8	31.1	– 123.1	72.0	30.8	– 155.0	1.13	63.0	28.7	– 113.6	66.08	30.7	– 150.6	1.05

Data are shown as median (10th – 90th range). Fold changes (FC) were calculated using the median. ***$P < 0.001$; **$P < 0.01$; *$P < 0.05$. Data were log-transformed and adjusted for age and BMI for statistical analyses. BMI: Body mass index.

Supplementary Table 5. **Risk of EC recurrence in relation to levels of 11-oxygenated androgens.**

	**Preoperative**					**Postoperative**				
**Steroids (pg/mL)**	LR *P*	HR_{adj}	*P*	HR_{Fadj}	*P*	LR *P*	HR_{adj}	*P*	HR_{Fadj}	*P*
**Free**										
11OHA4	0.98	0.97 (0.45–2.09)	0.94	1.15 (0.50–2.63)	0.74	0.77	1.05 (0.46–2.41)	0.92	0.94 (0.35–2.52)	0.90
11KA4	0.80	0.98 (0.44–2.17)	0.96	1.09 (0.47–2.55)	0.84	0.34	1.39 (0.59–3.24)	0.45	1.13 (0.42–3.03)	0.82
11OHT	0.96	1.04 (0.48–2.29)	0.92	0.96 (0.40–2.29)	0.93	0.78	0.81 (0.34–1.95)	0.64	1.15 (0.41–3.19)	0.79
11KT	0.71	0.66 (0.30–1.49)	0.32	0.66 (0.27–1.58)	0.35	0.80	1.11 (0.47–2.60)	0.81	1.04 (0.38–2.87)	0.94
11OHAST	**0.04**	2.14 (0.92–4.99)	0.08	2.24 (0.89–5.60)	0.09	0.05	2.25 (0.90–5.61)	0.08	3.23 (1.11–9.40)	**0.03**
11KAST	**0.005**	3.01 (1.20–7.52)	**0.02**	2.99 (1.09–8.18)	**0.03**	0.79	0.99 (0.40–2.44)	0.99	1.42 (0.50–4.07)	0.51
**Total**										
11OHA4	0.66	1.19 (0.55–2.53)	0.66	1.28 (0.55–2.98)	0.57	0.47	1.28 (0.56–2.93)	0.56	1.08 (0.39–2.98)	0.88
11KA4	0.05	1.67 (0.72–3.87)	0.23	2.41 (0.93–6.12)	0.07	0.31	1.34 (0.56–3.21)	0.52	1.38 (0.50–3.77)	0.53
11OHT	0.61	1.56 (0.71–3.41)	0.27	1.60 (0.67–3.81)	0.29	0.80	0.90 (0.37–2.20)	0.82	1.26 (0.45–3.53)	0.66
11KT	0.41	0.61 (0.28–1.36)	0.23	0.61 (0.26–1.43)	0.26	0.11	0.58 (0.24–1.40)	0.23	0.56 (0.19–1.64)	0.29
11OHAST	0.17	0.48 (0.22–1.05)	0.07	0.50 (0.22–1.14)	0.10	0.30	1.35 (0.56–3.24)	0.50	1.93 (0.72–5.18)	0.19
11KAST	0.53	1.46 (0.66–3.25)	0.35	1.40 (0.60–3.26)	0.44	0.71	1.06 (0.45–2.51)	0.89	1.07 (0.39–2.99)	0.89
SHBG (nmol/L)	0.09	1.56 (0.64–3.83)	0.33	/		0.28	1.50 (0.55–4.08)	0.42	/	

LR *P*: Log-rank *P* from Kaplan-Meier analysis for all available follow-up.
HR_{adj}: Hazard ratio and 95% confidence interval (95% CI), calculated with Cox regression for all available follow-up and adjusted for age, BMI, histological type and myometrial invasion. HR_{Fadj}: Cox regression was calculated as HR_{adj} and further adjusted for SHBG levels (Fadj = fully adjusted model). In the analysis restricted to hazard ratio 5-year follow-up after surgery, results were similar.

Supplementary Table 6. Disease-free survival (DFS) in relation to 11-oxygenated androgens

	**Preoperative**					**Postoperative**				
**Steroids (pg/mL)**	**LR *P***	**HR_{adj}**	***P***	**HR_{Fadj}**	***P***	**LR *P***	**HR_{adj}**	***P***	**HR_{Fadj}**	***P***
**Free**										
11OHA4	0.55	1.25 (0.66–2.40)	0.49	1.40 (0.71–2.76)	0.33	0.90	0.76 (0.36–1.60)	0.47	0.60 (0.28–1.43)	0.36
11KA4	0.41	0.87 (0.47–1.63)	0.67	0.88 (0.46–1.69)	0.70	0.93	0.98 (0.49–1.97)	0.96	0.96 (0.43–2.14)	0.92
11OHT	0.46	0.97 (0.50–1.89)	0.92	0.87 (0.43–1.76)	0.70	0.79	1.18 (0.57–2.48)	0.65	1.43 (0.61–3.33)	0.41
11KT	0.69	0.96 (0.52–1.79)	0.91	0.89 (0.46–1.73)	0.73	0.68	1.41 (0.69–2.91)	0.35	1.31 (0.57–2.97)	0.52
11OHAST	0.12	1.62 (0.85–3.10)	0.15	1.68 (0.85–3.32)	0.13	**0.02**	2.46 (1.13–5.34)	**0.02**	3.27 (1.34–8.00)	**0.009**
11KAST	0.20	1.67 (0.88–3.17)	0.12	1.50 (0.77–2.93)	0.23	0.64	0.92 (0.42–2.03)	0.83	1.14 (0.46–2.83)	0.77
**Total**										
11OHA4	0.60	1.18 (0.64–2.21)	0.58	1.33 (0.69–2.56)	0.39	0.58	1.00 (0.49–2.03)	0.99	1.01 (0.42–2.40)	0.99
11KA4	0.35	1.40 (0.75–2.60)	0.29	1.59 (0.82–3.06)	0.17	0.71	1.15 (0.56–2.36)	0.71	1.37 (0.593–.15)	0.47
11OHT	0.74	1.55 (0.78–3.06)	0.21	1.64 (0.79–3.39)	0.19	0.98	1.04 (0.47–2.29)	0.92	1.15 (0.47–2.83)	0.76
11KT	0.52	0.96 (0.52–1.77)	0.90	0.90 (0.47–1.72)	0.75	0.35	0.82 (0.41–1.67)	0.59	0.81 (0.36–1.85)	0.62
11OHAST	0.33	0.73 (0.39–1.38)	0.33	0.80 (0.41–1.56)	0.52	0.42	1.36 (0.65–2.68)	0.45	1.66 (0.74–3.73)	0.22
11KAST	0.81	1.18 (0.63–2.23)	0.61	1.06 (0.55–2.05)	0.85	0.12	0.71 (0.34–1.49)	0.36	0.69 (0.29–1.64)	0.40
SHBG (nmol/L)	0.33	0.85 (0.51–1.42)	0.54	/		0.14	1.05 (0.37–2.96)	0.93	/	

All-cause mortality. LR *P*: Log-rank *P* from Kaplan-Meier analysis for all available follow-up information.
HR_{adj}: Hazard ratio and 95% confidence interval (95% CI), calculated with Cox regression for all available follow-up and adjusted for age, BMI, low/high risk categories, metastases, lymph-vascular space invasion (LVSI) and recurrence; Low/High risk: TI-G1/G2 are categorized as low risk, while TI-G3 and TII are high risk. HR_{Fadj}: Cox regression was calculated as HR_{adj} and further adjusted for SHBG levels (Fadj = fully adjusted model). In the analysis restricted to hazard ratio 5-year follow-up after surgery, results were similar.

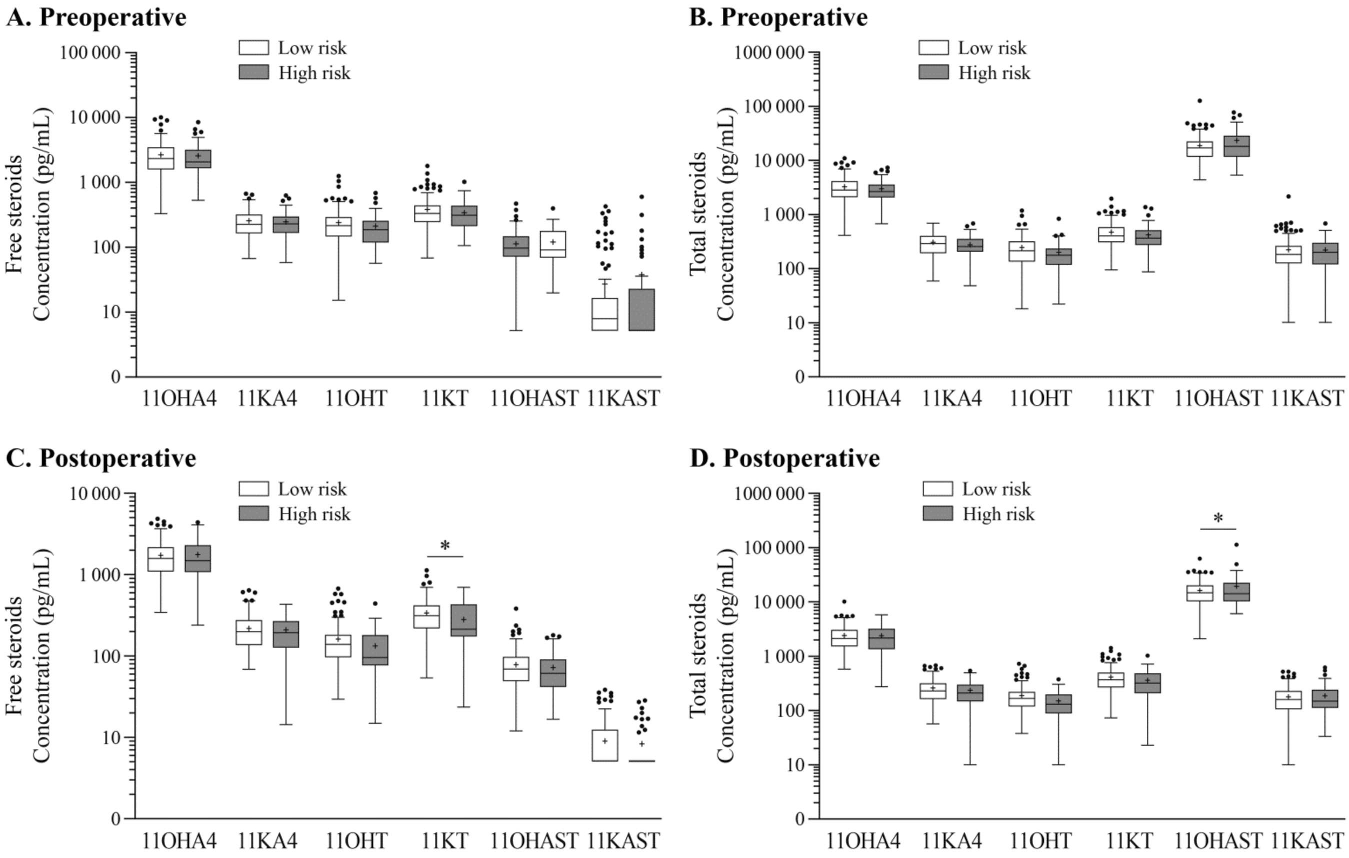

Supplementary Figure 1. Postoperative levels of 11-oxygenated androgens were associated with poor prognosis in endometrial cancer cases. Risk of poor prognosis was categorized as low risk corresponding to type I (TI) with low-grade G1 and G2 whereas TI-G3 and TII were considered as high risk. Box plot depicts 25-75 percentile and whiskers with the median shown as solid line and mean shown as + . Levels of 11-oxygenated androgens were log transformed and adjusted for age and BMI for statistics. * $P<0.05$. Total = free + conjugated (sulfates + glucuronides).

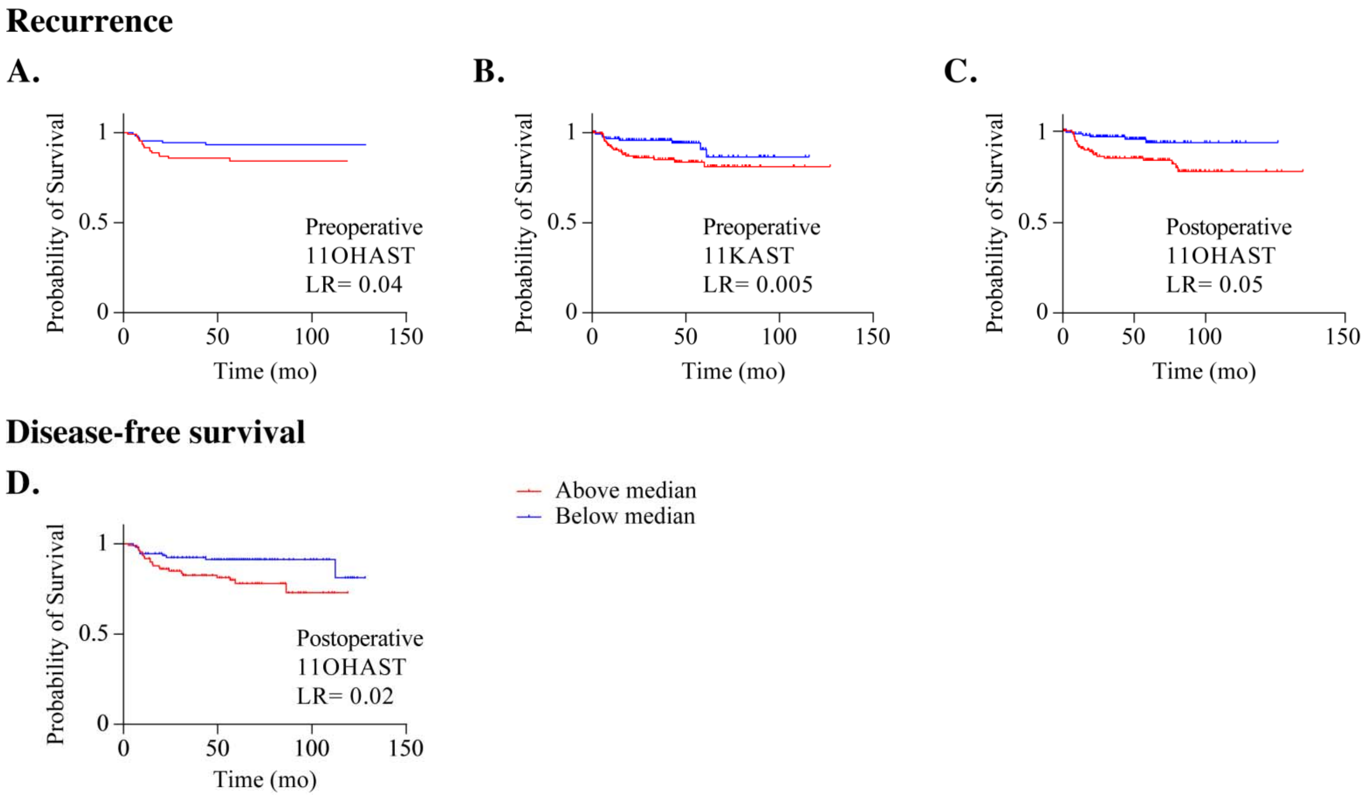

Supplementary Figure 2. Higher levels of 11-oxygenated androgens were associated with shorter time to recurrence and disease-free survival in EC cases. Kaplan-Meier survival curves are shown for time to recurrence (A, B, C) and disease-free survival (D). Log-rank test was used to determine differences.
